# Patient preferences for heart valve disease intervention

**DOI:** 10.1111/hex.13929

**Published:** 2023-12-04

**Authors:** Simon Fifer, Brittany Keen, Polo Guilbert‐Wright, Kaoru Yamabe, Dale J. Murdoch

**Affiliations:** ^1^ Community and Patient Preference Research (CaPPRe) Sydney New South Wales Australia; ^2^ Edwards Lifesciences ANZ Sydney New South Wales Australia; ^3^ Graduate School of Public Policy The University of Tokyo Tokyo Japan; ^4^ The Prince Charles Hospital Brisbane Queensland Australia; ^5^ Faculty of Medicine The University of Queensland Brisbane Queensland Australia

**Keywords:** discrete choice experiment, heart valve disease, patient preference

## Abstract

**Background:**

This study aimed to determine how patients trade‐off the benefits and risks of two different types of procedures used to treat heart valve disease (HVD). It also aimed to determine patients' preferences for HVD treatments (predicted uptake) and the relative importance of each treatment attribute.

**Methods:**

A discrete choice experiment (DCE) was conducted in Australia and Japan with patients who required a heart valve procedure. Patients were stratified into three categories: no prior procedure experience, minimally invasive procedure experience and invasive procedure experience. DCE attributes included risk of mortality; risk of stroke; needing dialysis; needing a new pacemaker; valve durability; independence 1 month after surgery; and out‐of‐pocket expenses. Participants chose between two hypothetical labelled approaches to therapy (‘invasive procedure’ and ‘minimally invasive procedure’), with a separate opt‐out included. A mixed multinomial logit model was used to analyse preferences.

**Results:**

The DCE was completed by 143 Australian and 206 Japanese patients. Both populations demonstrated an overall preference for the minimally invasive procedure over the invasive procedure. All attributes tested significantly predicted choice and were important to patient decision‐making. However, patients' choices were most influenced by the durability of the valve and the likelihood of independence postprocedure, irrespective of their prior procedure experience. Differences in preference were observed between Australian and Japanese patients; valve durability was the most important attribute among Australian patients, while Japanese patients emphasised regaining independence postsurgery. Risk of mortality was less important relative to other key attributes in Japan; however, it remained significant to the model.

**Conclusions:**

HVD patients prefer a minimally invasive procedure over an invasive procedure, irrespective of prior treatment experience. Key attributes contributing to treatment preferences are valve durability and faster recovery. These results can be used to help inform healthcare decision‐makers about what features of heart valve procedures patients value most.

**Patient and Public Contribution:**

People with lived experience of HVD were included in multiple stages of the design phase of this research. First, patients and doctors were consulted by taking part in qualitative interviews. The qualitative interviews helped inform which treatment attributes to include in the DCE based on what was important to those with lived experience and those who help make treatment decisions on behalf of patients. Qualitative interview participants also assisted with the framing of questions in the online survey to ensure the terminology was patient‐friendly and relevant to those with lived experience. Following qualitative interviews, the DCE attribute list was agreed on in expert consultation with a steering committee, which included patient representatives and treating physicians (interventional cardiologists, cardiothoracic surgeons). The survey was also pilot tested with a small sample of patients and minor adjustments were made to the wording to ensure it was appropriate and meaningful to those with lived experience of HVD.

## 1 INTRODUCTION

Heart valve disease (HVD) is a broad term used to describe diseases and conditions affecting the heart's valves.^1^ In Australia, an estimated 500,000–600,000 people had HVD in 2021, and an estimated 254,000 people are living with undiagnosed HVD.^1^ In Japan, HVD has an estimated prevalence of 8.3 males per 100,000) and 12.4 females per 100,000 in 2015.^2^

Types of HVD include aortic stenosis (AS), mitral valve regurgitation, tricuspid valve regurgitation and atresia.^1^ AS is the most common HVD in Australia, present in 3% of people aged >65 years.^1^, ^3^ AS is also the most common HVD in Japan, with an estimated 1,200,000 people in 2017. However, degenerative mitral valve disease is almost as prevalent in Japan, with an estimated 1,040,000 people.^4^ Although the survival rate in asymptomatic AS patients is high, the mean overall survival rate is dramatically lower following symptom onset, with an estimated 2‐year mortality of 50%–68%.^3^ Between 2000 and 2020, the AS crude mortality rate increased in both Australia and Japan.^5^

Treatment of HVD has generally relied on surgery using procedures such as surgical aortic valve replacement (SAVR).^6^ More recently, less invasive procedures, such as transcatheter aortic valve implantation (TAVI), are becoming more common, particularly in patients with high or prohibitive surgical risk.^7^ Although TAVI is less established than SAVR, it is less invasive and associated with faster recovery and fewer complications,^6^, ^8^, ^9^ even among patients with intermediate or low surgical risk.^8^, ^10^ A systematic review and meta‐analysis of 8818 participants comparing SAVR and TAVI found that TAVI was associated with a significantly shorter hospital stay. However, neither technique was found to be superior since TAVI was found to reduce the risk of certain side effects while SAVR reduced the risk of others.^11^ This suggests that patients should be included in the process of shared decision‐making to pursue treatments that are in line with patients' goals, values and preferences.

Understanding the treatment preferences of people living with HVD is a cornerstone of shared decision‐making.^12^ Patient preferences in HVD treatments have previously been modelled. Patient preference modelling using adaptive swing weight elicitation demonstrated that patients with AS placed the highest value on attributes reflecting TAVI, including lower mortality, reduced invasiveness and quicker recovery time.^13^ People living with other HVDs also face similar decisions when choosing between catheter and open‐heart procedure; evidence from a discrete choice survey found that patients with mitral valve regurgitation preferred treatment attributes associated with transcatheter intervention over open‐heart surgery.^14^

Studies quantifying trade‐off decisions in the patient population for heart valve procedures are limited, particularly in Australia and Japan. Furthermore, earlier studies have identified significant heterogeneity amongst individuals' treatment preferences,^12^, ^13^ and little is known about the associations between patient characteristics and treatment choices.^13^ Hence, the objective of this study was to develop a greater understanding of patient preferences for HVD treatments within Australian and Japanese populations.

## 2 METHODS

The research approach, including the experimental design, followed good practice guidelines.^15^, ^16^ The study was approved by the Bellberry Limited Human Research Ethics Committee (study ref no. 2021‐02‐104‐A‐2) and the Japanese ethical review board, NPO‐MINS (approved in the 126th MINS Institutional Review Board Meeting, October 20, 2021). All participants provided electronic informed consent.

### 2.1 Participants

Patients were deemed eligible to participate in the study if they met the following criteria: Australian or Japanese citizenship or permanent residency; a diagnosis of HVD (AS, mitral or tricuspid valve regurgitation); HVD required treatment, either by previous treatment for HVD or considering treatment options or have a procedure scheduled to treat their HVD; not employed by a pharmaceutical or medical device company; aged ≥18 years (Australia) or ≥20 years (Japan). Participants were recruited via healthcare provider referrals, clinical trial recruitment agencies, online panels, social media marketing and patient advocacy organisations. All patients were compensated for their time and effort to take part in the study ($50 AUD/¥5,000 JPY gift card, panel points, or they could opt to donate this to a heart disease support group). Participants were grouped by their experience with heart valve procedures into (i) no procedure experience, (ii) invasive procedure experience, or (iii) minimally invasive procedure experience.

Japan and Australia were chosen as they exhibit mature health technology assessment markets. Furthermore, treatment rates in Japan appear to be low compared to other markets and undertaking this type of research helps to better understand the patient's perspective toward treatment.

### 2.2 Survey instrument development

The relative importance of each treatment attribute and benefit‐to‐risk trade‐off were assessed using a preference elicitation technique known as a discrete choice experiment (DCE). DCEs are a methodological approach to studying choice behaviour, with a theoretical background in psychology and economics,^17^, ^18^, ^19^, ^20^, ^21^ and are now commonly used to assess preferences in health.^22^ The DCE was incorporated as part of an online survey. The survey design and DCE attributes and levels were derived over multiple stages. The first stage was a rapid review of the literature, including preference studies, market research and clinical trials. Next, qualitative interviews were conducted with patients with HVD (five with AS in Australia, two with mitral valve and two with aortic valve disease in Japan) and doctors specialising in HVD (three interventional cardiologists and two cardiothoracic surgeons in Australia; one cardiologist and one cardiovascular surgeon in Japan). The DCE attribute list was agreed on in expert consultation with a steering committee comprising interventional cardiologists, cardiothoracic surgeons, patient representatives and representatives from the study sponsor, Edwards Lifesciences. The survey was piloted in each country before full recruitment efforts. Minor adjustments to the wording in the DCE were made for clarity before full launch. The Australian and Japanese surveys contained near‐identical DCEs, with only small changes made to the wording and cost information for local relevance.

The survey included questions on demographics, disease history, treatment, quality of life (QoL) and the DCE. The survey was piloted in Australia in July 2021 (*n* = 4 patients with AS) and in Japan in February 2022 (*n* = 8 patients with HVD). After minor adjustments to wording for clarity, the survey was piloted with another eight HVD patients in Japan. Minor adjustments to the wording in the DCE were made following pilot runs. All patients who participated in the pilot surveys were considered for the final sample.

### 2.3 DCE

The DCE was a labelled design presented to participants as a series of choice tasks. Each choice task represented a scenario showing two hypothetical treatment alternatives (one labelled as ‘invasive procedure’ and one labelled ‘minimally invasive procedure’) and an opt‐out alternative (‘neither of these procedures’). Participants were provided with the descriptions of the two treatment alternatives:

**Table:** 

Invasive procedure	Surgical valve repair/replacement (SVR) or open‐heart surgery is an invasive procedure. A cut of 8–10 in. long is made in your chest and your chest is opened to access your heart. Then, your heart is stopped while a machine takes over your heart and lung function. Stopping the heart allows the surgeon to see the valve more easily. An incision is made in the heart, so the surgeon can repair the damaged valve. Your heart is started again and your chest is stitched closed. The procedure normally takes 3–5 hours and you will likely go to an intensive care unit (ICU) for up to 2 days. You will be required to stay 1–3 weeks in the hospital after the procedure. Open‐heart surgery has been clinically available for over 15 years.
Minimally invasive procedure	Catheter valve repair/implantation is a minimally invasive procedure requiring a small incision to be made near your groin, and then a valve is inserted and guided to your heart using a long tube through an artery. The tube is used to implant a new valve in the heart to replace the diseased valve or repair the existing one. The duration of this procedure is 35–60 min. You will be required to stay 2–3 days in the hospital after the procedure. The catheter procedure has been clinically available for less than 13 years.

**Figure 1:**
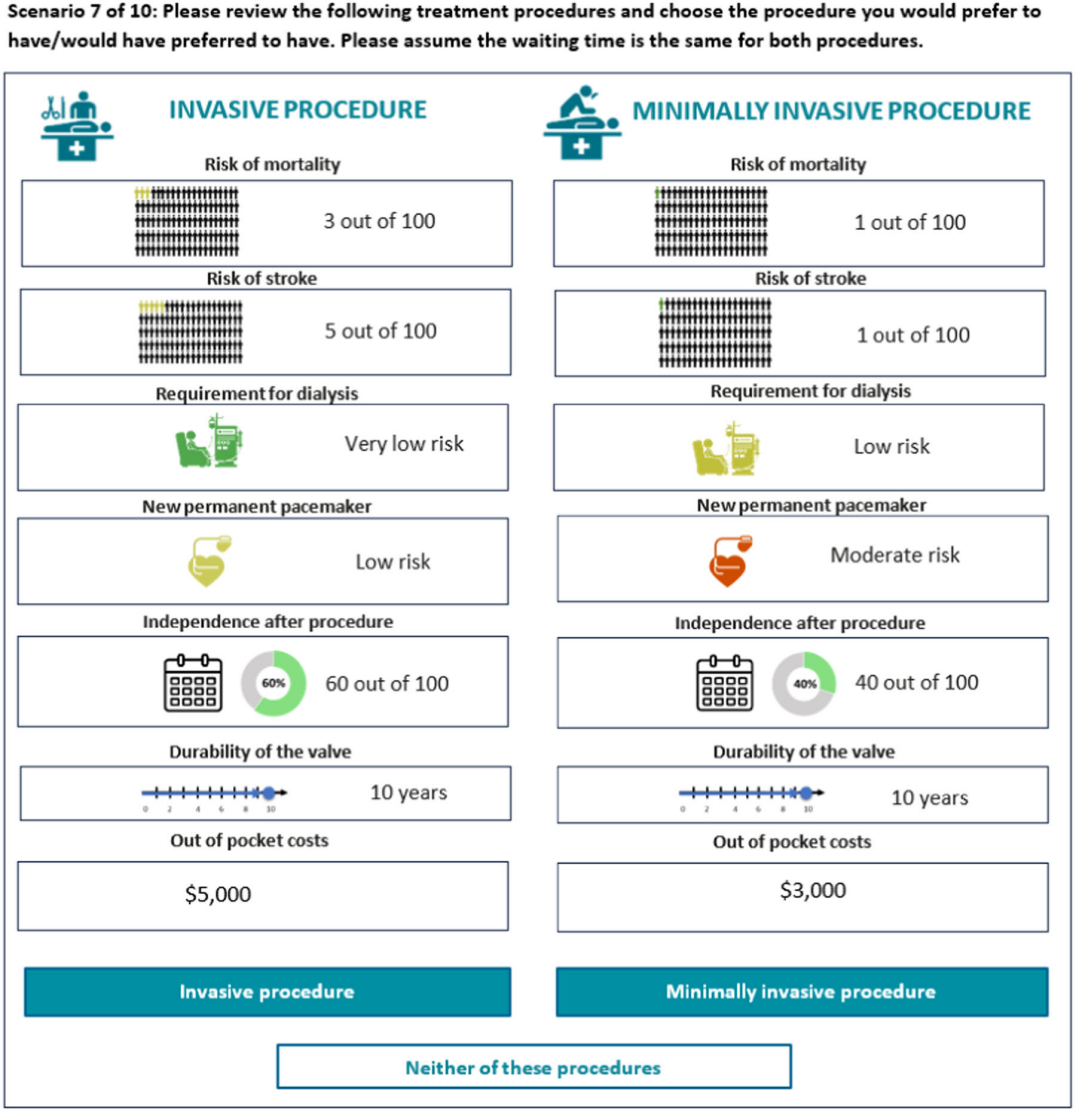
An example discrete choice experiment question presented to participants in Australia.

Participants were asked to choose which alternative they preferred according to their own value framework. An example of a patient choice scenario is shown in Figure 1. Hypothetical treatment alternatives were described by levels of the following attributes: risk of (i) mortality; (ii) stroke; (iii) needing dialysis; (iv) needing a new pacemaker; valve durability; independence after surgery; and out‐of‐pocket expenses. The detailed DCE grid describing the attributes and levels presented in the survey can be found in Table 1. Levels for each attribute systematically varied across the displayed hypothetical treatment alternatives. The DCE design consisted of 96 scenarios, split into eight blocks so that each participant was presented with 12 scenarios. Whenever the opt‐out was selected, participants were asked which of the two hypothetical treatment alternatives they preferred if they had to choose (forced choice). Forced choice data was used to cross‐check and validate participants' responses across the choice task and was not included in the final analysis. Before the choice scenarios, participants were told to assume that the waiting time for both procedures was the same.

**Table 1:** DCE attributes and levels for Australian participants.

Attribute	Description	Invasive procedure	Minimally invasive procedure	SAVR real values^a^	TAVI real values (SAPIEN 3)^a^
Risk of mortality	This refers to the number of patients out of 100 who will die within 1 year of having the procedure. Death could be due to complications from the procedure, from complications of heart valve disease, or as a result of a disabling stroke.	1%	1%		1%
		3%	3%	3%	
		5%	5%		
		7%	7%		
Risk of stroke	This refers to the number of patients out of 100 who will have a nonfatal disabling stroke within 1 month of the procedure. If you experience a stroke, you will be hospitalised. If the stroke is severe, it may lead to temporary or permanent disability, such as paralysis, reduced mobility and problems with thinking, memory and speech.	1%	1%	1%	1%
		5%	5%		
		10%	10%		
		15%	15%		
Requirement for dialysis	This refers to the number of patients out of 100 who will experience kidney function damage and who will need dialysis as a result of the procedure. A machine is used to do the kidney's job of cleaning the blood. If you need dialysis, you will need to go to the hospital three times a week, with each visit lasting 4 h.	Moderate risk (15%)	Moderate risk (15%)		
		Low risk (5%)	Low risk (5%)	Low risk (5%)	
		Very low risk (<1%)	Very low risk (<1%)		Very low risk (<1%)
New permanent pacemaker	This refers to the risk of needing a pacemaker permanently fitted as a result of the procedure. A pacemaker is an electrically charged medical device. Your surgeon implants it under your skin to help manage irregular heart rhythm.	Moderate risk (15%)	Moderate risk (15%)		
		Low risk (5%)	Low risk (5%)	Low risk (5%)	Low risk (5%)
		Very low risk (1%)	Very low risk (1%)		
Durability of the valve	This refers to the durability of the device/valve being implanted into your heart.	5 years	5 years		
		10 years	10 years	10 years	10 years
		15 years	15 years		
		20 years	20 years		
Independence after procedure	The proportion of patients who will have relief from heart valve disease symptoms, experience fewer physical and social limitations and feel more able to care for themselves at 1 month following the procedure.	10%	10%		
		30%	30%	30%	
		50%	50%		
		70%	70%		70%
Out‐of‐pocket costs	This refers to the out‐of‐pocket costs of the procedure.	$0	$0		
		$1500	$1500		
		$3000	$3000		
		$4500	$4500	$4500	$4500
		$6000	$6000		
		$7500	$7500		
		$9000	$9000		
		$10,500	$10,500		

Abbreviations: DCE, discrete choice experiment; SAVR, surgical aortic valve replacement; TAVI, transcatheter aortic valve implantation.

^a^ Real values for TAVI/SAVR were provided by Edwards Lifesciences and derived from the PARTNER 2 and 3 clinical trials,^6^, ^9^, ^10^ MAGICApp research^23^ and a meta‐analysis of SAVR outcomes.^24^

### 2.4 Analysis

Respondents were removed from the final sample if they finished the survey unreasonably quickly, had duplicate internet protocol (IP) addresses or invalid or nonsensical open‐text responses. Respondents who admitted to a poor understanding of the DCE task (rating less than seven out of 10 on understanding) and/or chose the opt‐out in more than 10 out of the 12 scenarios presented were also removed from the final sample.

The combinations of levels presented in the DCE scenarios were designed in accordance with the ISPOR guidelines^25^ using a D‐efficient design^26^ and generated using NGene (ChoiceMetrics Pty Ltd., Sydney, Australia). During DCE analysis, the categorical attributes were recoded using simple effects coding, with one level as a reference category (for an attribute with l levels, l−1 new variables were created).

DCE preference data were analysed using the mixed multinomial logit (MMNL) model, using Econometric software Nlogit version 6 (Econometric Software Inc.).^27^, ^28^ Parameter coefficients were treated as random variables drawn from a prespecified (normal or triangular) distribution. All parameters were treated as categorical variables whereby a positive coefficient for an attribute level indicates that it is preferred over the corresponding mean of the other levels, whereas a negative coefficient suggests the reverse effect.

## 3 RESULTS

### 3.1 Patient demographics and disease characteristics

A total of 249 Australian and 360 Japanese respondents completed the online survey; 83 Australian and 12 Japanese respondents were removed from the final sample as they finished the survey too quickly; 20 Australian and 42 Japanese respondents were removed as they admitted to a poor understanding of the DCE; three Australian respondents who chose the opt‐out in more than 10/12 scenarios were removed; 78 Japanese respondents with duplicate IP addresses and 22 Japanese respondents whose open‐text responses were considered nonsensical were removed. The final total analysed sample consisted of *n* = 143 participants in Australia and *n* = 206 participants in Japan (Table 2). Australian and Japanese patients completed the online survey in a median time of 25 and 23 min, respectively.

**Table 2:** Patient demographic, disease history and treatment history.

Demographic	Australian patient respondents (*n* = 143)	Japanese patient respondents (*n* = 206)
	*n* (%)	*n* (%)
Gender		
Female	61 (42.7%)	82 (39.8%)
Male	81 (56.6%)	124 (60.2%)
Nonbinary/gender fluid	1 (0.7%)	0 (0%)
Age		
20–30 (Japan)		6 (2.9%)
18–30 (Australia)	6 (4.2%)	
31–40	11 (7.7%)	24 (11.7%)
41–50	12 (8.4%)	35 (17.0%)
51–60	24 (16.8%)	75 (36.4%)
61–70	43 (30.1%)	48 (23.3%)
71–80	35 (24.5%)	16 (7.8%)
81–90	10 (7.0%)	2 (1%)
91 or older	2 (1.4%)	0 (0%)
Area of residence/practice		
Metropolitan/city	101 (70.6%)	163 (79.1%)
Regional	36 (25.2%)	43 (20.9%)
Rural	6 (4.2%)	0 (0%)
Highest education level attained (Australia)		
Year 11 or below	24 (16.8%)	
Year 12	15 (10.5%)	
Certificate III/IV	25 (17.5%)	
Bachelor's degree	35 (24.5%)	
Graduate diploma/graduate certificate	26 (18.2%)	
Masters or PhD	15 (10.5%)	
Prefer not to answer	3 (2.1%)	
Highest education level attained (Japan)		
Junior high school graduate		4 (1.9%)
High school graduates		32 (15.5%)
Vocational school graduates		18 (8.7%)
University graduates		120 (58.3%)
University graduate school (Master's or Doctoral)		31 (15.0%)
Prefer not to answer		1 (0.5%)
Occupation		
Working full time	45 (31.5%)	113 (54.9%)
Working part‐time	15 (10.5%)	11 (5.3%)
Working casual	8 (5.6%)	8 (3.9%)
Not working	4 (2.8%)	15 (7.3%)
Home duties and/or caring responsibilities	8 (5.6%)	12 (5.8%)
Retired	61 (42.7%)	40 (19.4%)
Other	2 (1.4%)	3 (1.5%)
Prefer not to answer	0 (0%)	4 (1.9%)
Treatment background		
No procedure experience	56 (39.2%)	11 (5.3%)
Minimally invasive procedure experience	39 (27.3%)	87 (42.2%)
Invasive procedure experience	48 (33.6%)	135 (65.5%)
Disease background^a^		
Aortic stenosis	75 (52.4%)	134 (65.0%)
Mitral valve regurgitation/disease	67 (46.9%)	98 (47.6%)
Tricuspid valve regurgitation/disease	38 (26.6%)	66 (32.0%)
Symptoms		
Mild	68 (47.6%)	109 (53%)
Moderate to severe	54 (37.8%)	50 (24%)
No symptoms	21 (14.7%)	47 (23%)

*Note*: Percentages may not add up to 100% due to rounding.

^a^ Participants were able to report more than one type of heart valve diagnosis.

### 3.2 Patient DCE–MMNL model results

DCE data were modelled separately for Australia and Japan (rather than pooled) due to the difference in the cost attribute levels and coding structures for some of the attributes (i.e., independence and risk of stroke). Comparisons between countries can still be made by comparing relative attribute importance and predicted treatment uptake. Random parameters were drawn from a normal distribution for Australia and a normal distribution for Japan (except for cost, which was drawn from a triangular distribution).

The results from the best‐fitting MMNL model are shown in Table 3. The parameter coefficients represent preference for one attribute level over the others *within* that attribute, when all else is held constant (to compare *across* attributes, see Sections 3.3 and 3.4). In both Australia and Japan, patients preferred heart valve procedures with the longest valve durability (Australia 20 years, *β* = .972, *p* ≤ .01; Japan 20 years, *β* = .372, *p* ≤ .01).

**Table 3:** Mixed multinomial logit model results for Australian and Japanese participants.

Random parameter	Australian participants				Japanese participants			
	Coefficient	Sig.	SE	*T* ratio	Coefficient	Sig.	SE	*T* ratio
Risk of mortality								
1%	0.571	***	0.142	4.030	0.457	***	0.109	4.200
3%	0.331	**	0.141	2.360	−0.103		0.085	−1.200
5%	−0.011		0.137	−0.080	−0.060		0.078	−0.780
*RC: 7%*	−0.891				−0.294			
Risk of stroke								
1%–5% (Japan only)					0.470	***	0.075	6.240
1% (Australia only)	0.537	***	0.143	3.760				
5% (Australia only)	0.254	**	0.128	1.990				
10%	0.018		0.140	0.130	−0.154	*	0.08	−1.920
*RC: 15%*	−0.809				−0.316			
Durability of valve								
10 years	−0.382	***	0.128	−2.990	−0.137		0.084	−1.620
15 years	0.478	***	0.142	3.370	0.156	*	0.083	1.880
20 years	0.972	***	0.182	5.330	0.372	***	0.093	3.990
*RC: 5 years*	−1.068				−0.392			
Independence after the procedure								
30%	−0.105		0.123	−0.860	−0.149	*	0.081	−1.840
50%–70% (Australia only)	0.861	***	0.141	6.090				
50% (Japan only)					0.323	***	0.093	3.480
70%					0.346	***	0.103	3.350
*RC: 10%*	−0.756				−0.519			
Requirement for dialysis								
Low risk	0.126		0.109	1.160	0.138	*	0.082	1.680
Very low risk	0.362	***	0.127	2.850	0.289	***	0.08	3.610
*RC: Moderate risk*	−0.488				−0.427			
New permanent pacemaker								
Low risk	0.039		0.114	0.340	−0.120	*	0.065	−1.850
Very low risk	0.368	***	0.111	3.330	0.254	***	0.071	3.580
*RC: Moderate risk*	−0.407				−0.134			
Cost								
Australia								
Low cost ($0, $1500)	0.531	***	0.162	3.260				
Medium cost ($3000, $4500, $6000)	0.304	**	0.137	2.210				
Medium/high cost ($7500, $9000)	−0.113		0.137	−0.830				
*RC: High cost ($10,500)*	−0.722							
Japan								
Low cost (¥50,000–¥100,000)					0.089		0.067	1.300
High cost (¥150,000–¥250,000)					−0.123	*	0.073	−1.680
*RC: No cost (¥0)*					0.017			
Alternative specific constant								
Invasive	3.399	***	0.339	10.020	2.935	***	0.201	14.570
Minimally invasive	3.714	***	0.348	10.660	2.876	***	0.192	15.010
*RC: Opt‐out (none of these)*								

*Note*: Australian participants: Log‐likelihood function: −1010.46974; restricted log‐likelihood:−1885.21869; McFadden pseudo *R* ^2^: 0.4640040; observations: 1716; respondents: 143. Japanese participants: Log‐likelihood function: −1733.76089; restricted log‐likelihood: −2715.76958; McFadden pseudo *R* ^2^: 0.3615950; observations: 2472; respondents: 206.

Abbreviations: SE, standard error; Sig, significance; RC, simple effects contrast coding reference category.

*, ** and *** denote significance at 10%, 5% and 1% levels, respectively.

There was a strong preference for a 50%–70% chance of independence 1 month after the procedure by both Australian (*β* = .861, *p* ≤ .01) and Japanese (50% *β* = .323, *p* ≤ .01; 70% *β* = .346, *p* ≤ .01) participants.

A lower risk of stroke (1%–5%) was preferred by both Australian and Japanese participants (*p* ≤ .05).

In both Australia and Japan, patients preferred a 1% risk of mortality (Australia *β* = .571; Japan *β* = .457; both *p* ≤ .01), very low risk of the need for dialysis (Australia *β* = .362, *p* ≤ .01; Japan *β* = .289, *p* ≤ .01) and a new pacemaker (Australia *β* = .368, *p* ≤ .01; Japan *β* = .254, *p* ≤ .01).

In both Australia and Japan, the significant positive *β* coefficients for the alternative‐specific constants suggest that patients preferred either heart valve procedure over the opt‐out (neither procedure) (Australia invasive treatment *β* = 3.399, *p* ≤ .01; minimally invasive procedure *β* = 3.714, *p* ≤ .01; Japan invasive treatment *β* = 2.935, *p* ≤ .01; minimally invasive procedure *β* = 2.876, *p* ≤ .01).

Australian participants showed a significant preference for a low (*β* = .531, *p* ≤ .01) or medium cost procedure (*β* = .304, *p* ≤ .05), with the greatest relative utility for the low‐cost procedure. Low‐ and medium‐cost procedures did not significantly predict choice for Japanese participants (*p* ≥ .05); however, high‐cost procedures were associated with a significant negative coefficient (disutility).

### 3.3 Relative attribute importance

**Figure 2:**
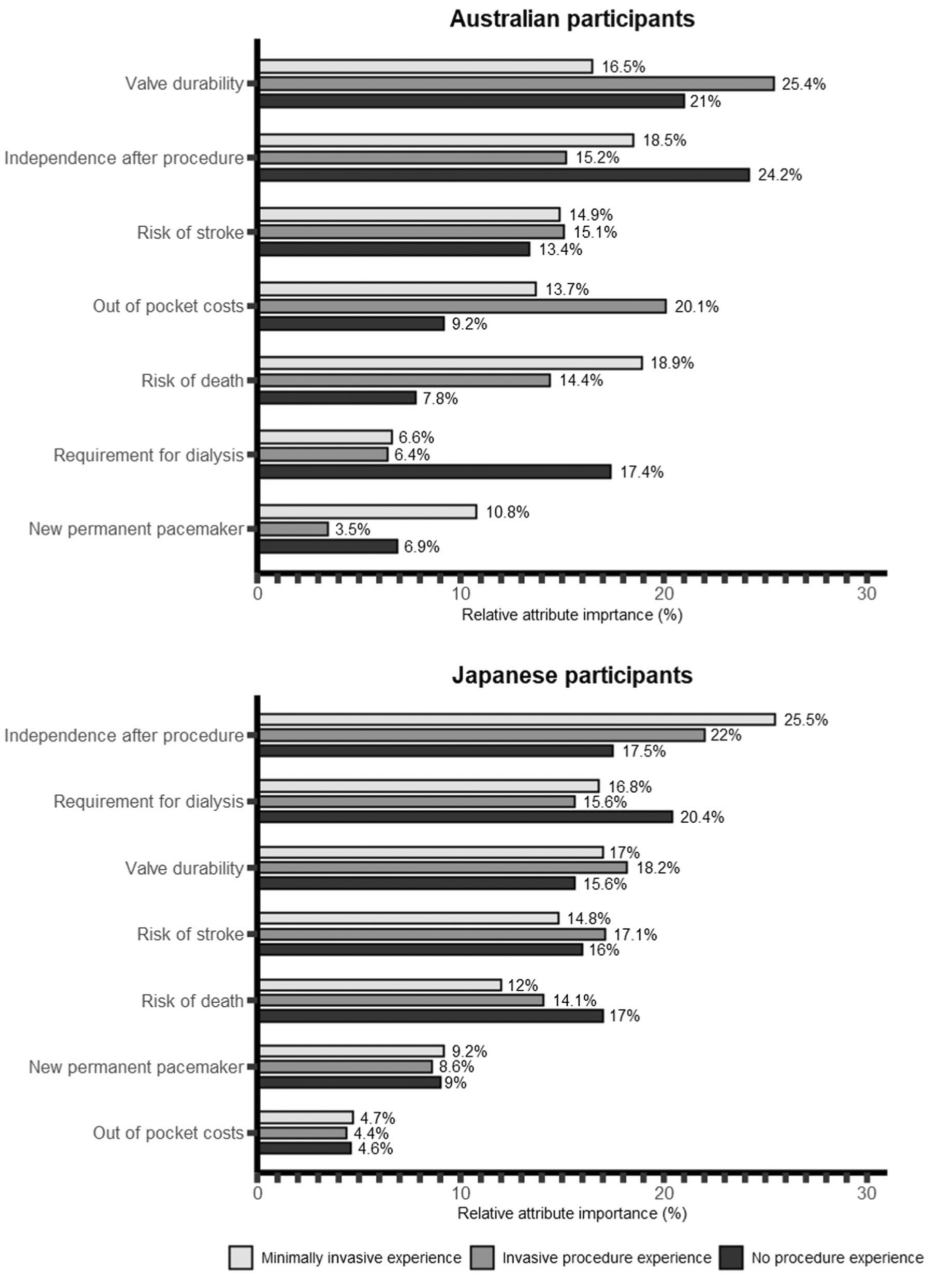
Attribute importance by patient procedure experience.

To complement the parameter estimates, relative attribute importance provides the opportunity to compare *across* attributes. The relative importance of each attribute represents the maximum difference in utility between the attribute's levels expressed as a percentage of the sum of all maximum differences.^29^ The relative attribute importance for patients with invasive procedure experience, minimally invasive procedure experience and no procedure experience is shown in Figure 2. Valve durability and independence after the procedure contributed greatly to treatment choices in both the Australian and Japanese populations. For example, for Australian patients with no prior procedure experience, improving the likelihood of regaining independence 1 month after the procedure was nearly three times as important as incremental changes to the risk of death in influencing treatment choice (relative attribute importance of independence = 24.2%; death = 7.8%). Australian participants who had undergone a minimally invasive procedure were less influenced by durability of the valve compared to those who had undergone an invasive procedure (16.5% vs. 25.4% relative attribute importance, respectively). Conversely, Japanese participants who had undergone a minimally invasive procedure were similarly influenced by valve durability than those who had undergone an invasive procedure (17% vs. 18.2%, respectively). Independence after the procedure was more important to Japanese participants than to Australian participants who have had a procedure previously. Japanese participants with any prior procedure experience placed greater importance on the potential need for future dialysis (15.6%–16.8%) compared to their Australian counterparts (6.4%–6.6%). The relative importance of out‐of‐pocket cost was greater to Australian participants (9.2–20.1%) than Japanese participants (4.4–4.7%), irrespective of prior treatment experience. This is most likely due to the range of the cost levels used in the experiment for each country.

### 3.4 Predicted procedure uptake by treatment experience

**Figure 3:**
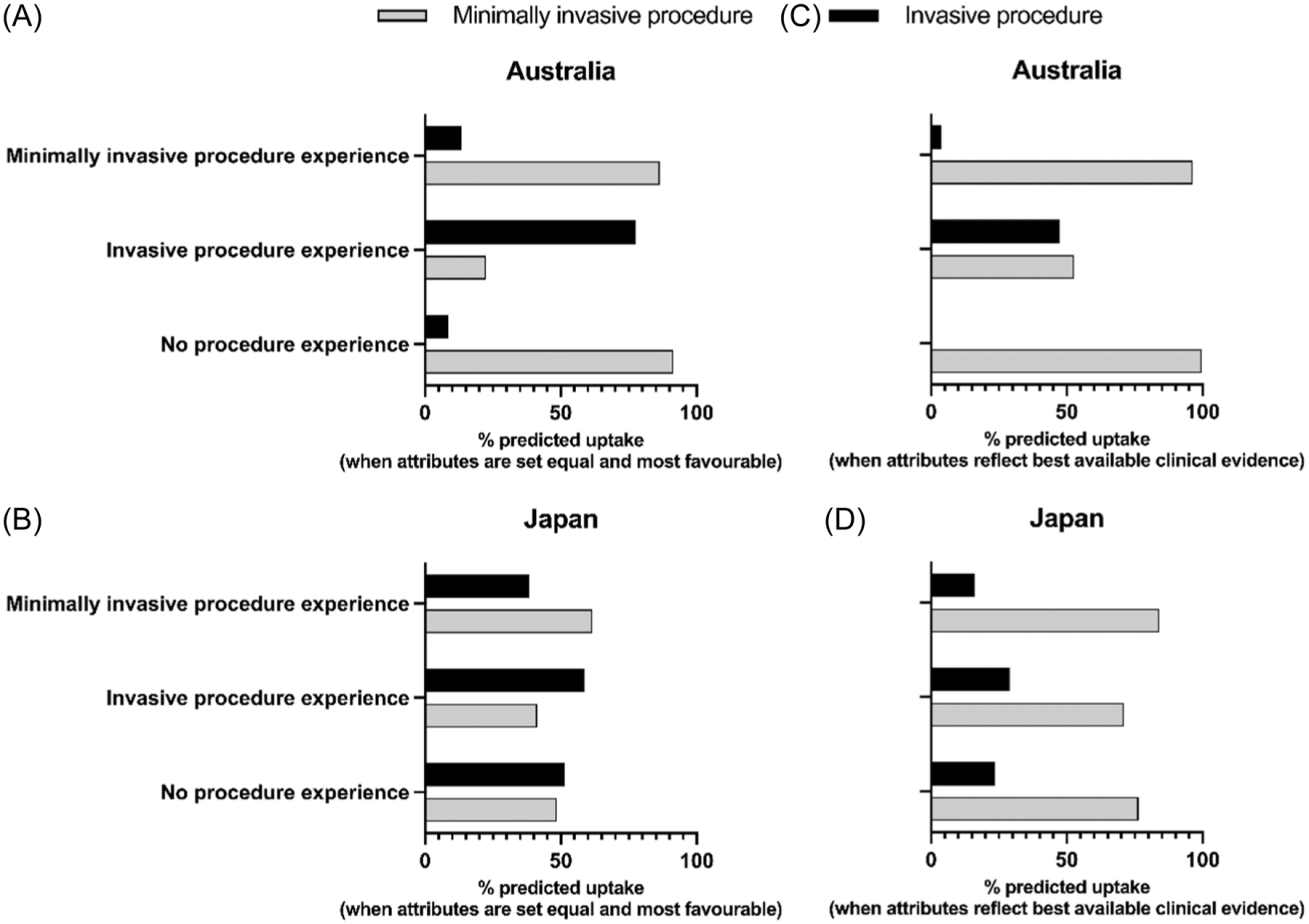
Predicted uptake for heart valve disease procedure by treatment experience when attributes are set equal and most favourable (A, B) or when attributes reflect best available clinical evidence for TAVI and SAVR (C, D). SAVR, surgical aortic valve replacement; TAVI, transcatheter aortic valve implantation.

Figure 3 demonstrates the predicted uptake for each procedure in each country by treatment experience. When all attributes in each treatment are held equal and most favourable (Figure 3A,B; which is a reflection of the strength of the influence of invasiveness of treatment, as represented by the model constants) more than 86% of Australian participants with either no procedure experience or minimally invasive procedure experience are predicted to choose the minimally invasive procedure (Figure 3A). Conversely, only 77.64% of Australian participants who previously had the invasive procedure are predicted to choose it again (Figure 3A). This suggests a small but notable ‘experience effect’ whereby patients are more likely to choose the same procedure they have already had, albeit slightly less so for those with invasive procedure experience (who are more likely to switch treatments compared to those with minimally invasive procedure experience). Japanese participants also appeared to be influenced by their past treatment decisions, albeit not to the same extent as the Australian patients (Figure 3B). Nearly 62% of Japanese patients who had previously undergone a minimally invasive treatment are predicted to choose it again, and 59% of those with invasive procedure experience are predicted to choose it again.

Notably, when attributes reflect best available clinical evidence for TAVI versus SAVR, it is predicted that the majority of people, irrespective of nationality or previous procedure experience, will choose the minimally invasive procedure (Figure 3C,D); predicted uptake increases between 72% and 100% for the minimally invasive procedure in all groups with the exception of Australians with invasive procedure experience, for whom there is only a small, but majority preference (52.74%) for the minimally invasive procedure, further demonstrating the power of past treatment experience on future treatment decisions. However, in Australia, predicted uptake for the invasive procedure under these values is between 4% and 47%. In Japan, predicted uptake for the invasive procedure ranges between 16% and 29%, whereas uptake for the minimally invasive procedure ranges between 71% and 84%.

## 4 DISCUSSION

The current study found an overall preference for minimally invasive procedures over invasive procedures. These findings affirm and expand on previous choice‐based research that reported a preference for minimally invasive procedures and independence following treatment.^13^, ^14^ All attributes tested in this study significantly predicted choice and were important to patient decision‐making. However, patients' choices were most influenced by the durability of the valve and the likelihood of independence after the procedure, irrespective of their previous procedure experience.

Cultural and regional influences may play a role in patient preferences for heart valve treatment. When making decisions in the choice task, valve durability was the most important attribute for Australian patients, whilst Japanese patients placed the greatest emphasis on the chance of regaining their independence 1 month after surgery. Australian patient choices were also more influenced by out‐of‐pocket costs than their Japanese counterparts. Moreover, the risk of mortality was less important relative to other key attributes in Japan, although it still remained significant to the model.

Based on the study's findings, it is predicted that the majority of people, irrespective of nationality or prior procedure experience, would prefer a minimally invasive procedure over an invasive procedure.

When all treatment attributes were held constant and we isolated the effect of invasiveness of the procedure, patients in Australia and Japan were more likely to choose the treatment they had already had experience with (an ‘experience effect’). For those who had not yet had treatment, Australians were more likely to choose the minimally invasive procedure and Japanese patients were roughly evenly split. Importantly, the preference for the TAVI treatment profile was so strong that it reversed the influence of prior treatment experience, and all patient groups in both countries were predicted to choose TAVI over the SAVR profile. This is an important finding and highlights that future DCEs should account for prior treatment experience when modelling the data. Although the disease burden of HVD is significant in both regions,^30^ preference for the minimally invasive procedure in Japan may be driven by a greater value on independence postprocedure (minimally invasive = 70% vs. invasive = 30%). Previous research was unable to examine the influence of treatment experience on HVD treatment choice, either due to sample size constraints^13^ or a lack of stratification according to invasiveness of past procedures.^14^ However, it has been reported that age can contribute to the interplay between prior experience and the value of valve durability.^14^ A previous study showed that patients who undergo open‐heart surgery are a relatively younger cohort, meaning valve longevity possesses greater utility for treatment outcomes.^12^ Another influencing factor reported may be patient expectations, as some patients may perceive less invasive procedures as less durable than surgical interventions.^7^, ^12^

Study interpretation should be considered in light of some important limitations. Neither the patients in Australia nor in Japan were recruited through a random approach and so representativeness of the general HVD population cannot be assured. To recruit a participant pool large enough for the planned analyses, the study population was not exclusively comprised of a single type of HVD, but rather multiple HVDs. Patients with various HVDs (AS and mitral/tricuspid valve regurgitation) were considered appropriate for the current study as they face similar treatment decisions when considering interventions.^14^, ^31^ As such, applying this study's findings to one particular intervention/comparison is limited. The sample size for the Japanese ‘no procedure experience’ cohort is small, hence small variations in patient preferences may skew the data. Diagnoses were self‐reported, as physician confirmation would not be feasible for the sample size in this study.

In conclusion, this study demonstrates that HVD patients generally prefer a minimally invasive procedure over an invasive procedure, irrespective of prior treatment experience. Key attributes contributing to patient preference when choosing between invasive versus minimally invasive procedures are valve durability and independence after the procedure. These findings provide a better understanding of treatment aspects that are most important to patients with HVD in Australia and Japan and can be used to help guide healthcare decision‐makers about what value patients place on features of heart valve procedures.

## AUTHOR CONTRIBUTIONS

**Simon Fifer**: Conceptualisation (equal); data curation (equal); formal analysis (lead); investigation (equal); methodology (lead); supervision (equal); writing—original draft preparation (equal); writing—review and editing (equal). **Brittany Keen**: Conceptualisation (supporting); data curation (equal); investigation (equal); methodology (supporting); project administration (lead); supervision (equal); writing—original draft preparation (equal); writing—review and editing (lead). **Polo Guilbert‐Wright**: Conceptualisation (equal); funding acquisition (lead); supervision (equal); writing—original draft preparation (equal); writing—review and editing (equal). **Kaoru Yamabe**: Writing—original draft preparation (equal); writing—review and editing (equal). **Dale Murdoch**: Conceptualisation (supporting); writing—original draft preparation (equal); writing—review and editing (equal)

## CONFLICT OF INTEREST STATEMENT

Brittany Keen and Simon Fifer are employees of CaPPRe. CaPPRe has consulted with Abbvie, Amgen, AstraZeneca, BMS, Celgene, CSL Behring, Edwards Lifesciences, GSK, Ipsen, Janssen, Medtronic, MSD, Novo Nordisk, Roche, Sanofi Sequiris, Takeda (Shire), UCB and Vertex outside of the submitted work. Dale J. Murdoch is a consultant and proctor for Edwards Lifesciences. Polo Guilbert‐Wright is an employee of Edwards Lifesciences. The remaining author declares no conflict of interest.

## Data Availability

The data that support the findings of this study are available on request from the corresponding author. The data are not publicly available due to privacy or ethical restrictions.
